# Development and validation of a genomic nomogram based on a ceRNA network for comprehensive analysis of obstructive sleep apnea

**DOI:** 10.3389/fgene.2023.1084552

**Published:** 2023-03-10

**Authors:** Wang Liu, Xishi Sun, Jiewen Huang, Jinjian Zhang, Zhengshi Liang, Jinru Zhu, Tao Chen, Yu Zeng, Min Peng, Xiongbin Li, Lijuan Zeng, Wei Lei, Junfen Cheng

**Affiliations:** ^1^ The Second Affiliated Hospital of Guangdong Medical University, Zhanjiang, China; ^2^ Affiliated Hospital of Guangdong Medical University, Zhanjiang, China

**Keywords:** obstructive sleep apnea, biomarkers, ceRNA, lncRNA, nomogram

## Abstract

**Objectives:** Some ceRNA associated with lncRNA have been considered as possible diagnostic and therapeutic biomarkers for obstructive sleep apnea (OSA). We intend to identify the potential hub genes for the development of OSA, which will provide a foundation for the study of the molecular mechanism underlying OSA and for the diagnosis and treatment of OSA.

**Methods:** We collected plasma samples from OSA patients and healthy controls for the detection of ceRNA using a chip. Based on the differential expression of lncRNA, we identified the target genes of miRNA that bind to lncRNAs. We then constructed lncRNA-related ceRNA networks, performed functional enrichment analysis and protein-protein interaction analysis, and performed internal and external validation of the expression levels of stable hub genes. Then, we conducted LASSO regression analysis on the stable hub genes, selected relatively significant genes to construct a simple and easy-to-use nomogram, validated the nomogram, and constructed the core ceRNA sub-network of key genes.

**Results:** We successfully identified 282 DElncRNAs and 380 DEmRNAs through differential analysis, and we constructed an OSA-related ceRNA network consisting of 292 miRNA-lncRNAs and 41 miRNA-mRNAs. Through PPI and hub gene selection, we obtained 7 additional robust hub genes, CCND2, WT1, E2F2, IRF1, BAZ2A, LAMC1, and DAB2. Using LASSO regression analysis, we created a nomogram with four predictors (CCND2, WT1, E2F2, and IRF1), and its area under the curve (AUC) is 1. Finally, we constructed a core ceRNA sub-network composed of 74 miRNA-lncRNA and 7 miRNA-mRNA nodes.

**Conclusion:** Our study provides a new foundation for elucidating the molecular mechanism of lncRNA in OSA and for diagnosing and treating OSA.

## 1 Introduction

Obstructive sleep apnea (OSA) is the repeated partial or complete collapse of the upper airway during sleep, resulting in intermittent hypoxemia, which influences the onset and progression of the disease (Duarte et al., 2020; Zhou et al., 2021). OSA may be associated with multiple system diseases over time, including hypertension, coronary heart disease, type 2 diabetes, cerebral infarction, Alzheimer’s disease, Parkinson’s disease, and non-alcoholic fatty liver disease (Floras, 2015; Salman et al., 2020; Zhou et al., 2021). OSA affects at least 2%–4% of the adult population, and the prevalence of OSA in patients aged 65 and older exceeds 30% (Ekin et al., 2021). In clinical practice, weight loss therapy, positive airway pressure ventilation therapy, surgical therapy, oral appliance therapy, and drug therapy are frequently used, but the results are not satisfactory (Kendzerska et al., 2021). High prevalence and ineffective treatment will have a negative impact on the quality of life of patients. Therefore, there is an urgent need to identify new biomarkers and treatment targets for OSA that are more effective.

Non-coding RNA (ncRNA) is a functional RNA molecule that is not translated into protein, such as microRNA (miRNA), long non-coding RNA (lncRNA), circular RNA (circRNA), intronic RNA, small interfering RNA (siRNA), small nucleolar RNA (snoRNA), and piwi-interacting RNA (piRNA), among others (Matsui and Corey, 2017). Long non-coding RNAs (lncRNAs) are a class of transcripts with a length of >200 nucleotides that are incapable of encoding proteins but play a crucial role in gene regulation, biological processes, and a variety of diseases (Zhou et al., 2021). Recent studies have found that lncRNA XIST promotes the occurrence and development of OSAHS by downregulating the expression of GRα in the adenoids of OSAHS children, which may provide a potential therapeutic target for OSAHS (Zhou et al., 2021). MicroRNA (miRNA) is a non-coding, single-stranded molecule of approximately 22 nucleotides that fine-tunes the expression of its target genes after transcription by interfering with the 3′-UTR region of mRNA (Haenisch et al., 2015; Li and Yuan, 2020). This interference results in mRNA degradation or inhibition of protein translation. Therefore, misregulation of miRNA will result in changes in protein expression, leading to disease development (Li and Yuan, 2020). Overexpression of miR-107 inhibits the expression of hypoxia-inducible factor 1 (HIF-1b) and hypoxia signaling [10] (Li et al., 2017); conversely, overexpression of miR-107 increases the expression of hypoxia-inducible factor 1 (HIF-1a) and hypoxia signaling.

There is evidence that the activities of lncRNA and miRNA are intertwined through a variety of complex mechanisms (Yamamura et al., 2018), as a result of the advancement of research. Among the mechanisms is the function of lncRNA as competing endogenous RNA (ceRNA), which compete with mRNAs for binding to miRNA binding sites, thereby negatively regulating miRNA and its target genes (Xu et al., 2021). In mice, cardiac apoptosis-related lncRNA CARL can acquire miR-539, which indirectly upregulates its target PHB2 and regulates apoptosis and mitochondrial fission (Rey et al., 2021). The highly expressed lncRNA-Adi in rat adipocytes interacts with miR-449a, which enhances the expression of the miRNA target CDK6, and then participates in the regulation of the formation of beige cellular tissue (Hou et al., 2018; Chen et al., 2020). The lncRNA MALAT1 can play a regulatory role by acting on miR-224-5p, thereby regulating the hippocampal NLRP3/IL-1β pathway and inhibiting the hippocampus inflammatory response in type 2 diabetic patients with OSA (Du et al., 2020).

In this study, plasma samples were collected from OSA patients and a normal control group, plasma RNA was extracted, and ceRNA chip detection was performed. Based on the differential expression of lncRNA, we identified the target genes of miRNA that bind to lncRNAs. We then constructed lncRNA-related ceRNA networks, performed functional enrichment analysis and protein-protein interaction analysis, and performed internal and external validation of the expression levels of stable hub genes. Then, we performed LASSO regression analysis on the stable hub genes, selected relatively significant genes to construct a simple and easy-to-use nomogram, validated the nomogram, and constructed the core ceRNA sub-network of key genes. This study identified potential target genes of miRNA that may be involved in the combination of lncRNA in OSA, providing a foundation for the study of the pathogenesis of OSA and the diagnosis and treatment of OSA.

## 2 Methods

### 2.1 Study subjects

In this study, 54 participants were recruited between December 2020 and May 2021. Patients who met the inclusion and exclusion criteria were selected, and the final sample consisted of 9 volunteers; 6 OSA patients and 3 healthy volunteers who served as the control group. All participants were subjected to a Watch-PAT examination (the specification model is Watch-PAT 200, and the manufacturer is Israel Ita), in addition to anthropometric measurements, blood pressure measurements, and blood biochemical tests. According to the inclusion and exclusion criteria, eligible patients were selected. Inclusion criteria: 1) The experimental group was comprised of volunteers whose Watch-PAT test result indicated OSA; 2) Patients aged between 28 and 36 years old (including 28 and 36 years old); 3) Patients with the capacity to act independently and consent to sign the informed consent form; and 4) Patients with a total sleep time >4 h5) Healthy volunteers were recruited for the control group. Exclusion criteria: 1) Patients with coronary heart disease, hypertension, diabetes, kidney disease, chronic pulmonary disease, or cerebrovascular disease; 2) patients with severe organ failure; 3) a history of brain tumors or epilepsy; 4) patients with various mental and psychological diseases who were taking sedatives and sleeping drugs; 5) OSA patients who had previously received treatment: The Medical Ethics Committee of the Second Affiliated Hospital of Guangdong Medical University approved this study (ethics number: GDEFEY2020LS030).

### 2.2 Basic data collection

In our study, we collected the patient’s name, age, gender, neck circumference, waist circumference, weight, height, and blood pressure. We then calculated the patient’s body mass index (BMI) using the formula: weight (kg)/height (m)^2^ (BMI = kg/m^2^). The researchers then modify the NoSAS score using general information. NoSAS (Marti-Soler et al., 2016) includes 5 questions: The first issue is that a neck circumference ≥40 cm is worth 4 points; the second issue is the BMI value range: 25 ≤ BMI <30 kg/m^2^ is worth 3 points, BMI ≥30 kg/m^2^ is worth 5 points, and snoring is worth 2 points. The answer to the fourth question is that age ≥55 is worth 4 points; the score for the fifth question for male patients is 2. If the NoSAS score is ≥ 8, it indicates that OSA patients are at high risk.

### 2.3 Watch-PAT detection

The primary function of the Watch-PAT sleep monitoring device is to detect sleep-disordered breathing. On the day of the examination, participants were instructed to abstain from alcohol, caffeine, and sleep aids. The Watch-PAT sleep monitoring device primarily monitors PAT, heart rate, blood oxygen saturation, snoring, body position, and additional sleep or waking stage parameters. The software analyzes the changes in the PAT signal throughout the entire sleep process. The sleep time of all patients should be monitored for at least 7 h. The diagnostic criteria for OSA were defined as apnea hypopnea index (AHI) ≥5 times/h, but the criteria were further subdivided into mild OSA (5 ≤ AHI <15 times/h), moderate OSA (15 ≤ AHI <30 times/h), and severe OSA (AHI ≥30 times/h).

### 2.4 Blood sample collection

Our study participants were divided into three groups: the normal group, the training cohort (obese OSA), and the internal validation cohort (non-obese OSA). There were three members in each group. All participants provided two blood samples at 8:00 a.m., following a full night of Watch-PAT sleep monitoring and overnight fasting. Then, we collected the blood into EDTA purple anticoagulant tubes; one was sent for blood glucose and blood lipid detection, while the other was used for ceRNA chip detection. Within 60 min of blood collection, the blood was centrifuged for 10 min at 3,000 g to separate plasma. The supernatant was transferred to an RNase-free Eppendorf tube and stored at −80°C until RNA extraction.

### 2.5 ceRNA expression profile

Total RNA was isolated using RNeasy Total RNA Isolation Kit (Qiagen, GmBH, Germany)/TRIzol reagent (Life technologies, Carlsbad, CA, United States) per the manufacturer’s instructions, purified using an RNeasy Mini Kit (Qiagen, GmBH, Germany), and quantified using Nanodrop. Using the Agilent Bioanalyzer 2,100 (Agilent technologies, Santa Clara, CA, United States), the fragment distribution of total RNA was analyzed. The RNA from each group was then used to generate biotinylated cRNA targets for the Sino Human ceRNA array V3.0. cRNA targets that were biotinylated were then hybridized with the slides. The Agilent Microarray Scanner was used to scan the slides after hybridization (Agilent technologies, Santa Clara, CA, United States). Using Feature Extraction software 10.7, data was extracted (Agilent technologies, Santa Clara, CA, United States). Quantile algorithm, R package “Limma” were used to normalize the raw data (Ritchie et al., 2015). At Sinotech Genomics Corporation, the microarray experiments were conducted according to the protocol developed by Agilent technologies Inc. Genes exhibiting a fold change of at least 1 were chosen for further examination.

### 2.6 Identification of differentially expressed lncRNA and mRNA between OSA group and normal group

The “limma” package in R is used to identify differentially expressed lncRNA and mRNA between OSA and normal groups, an efficient bioinformatics analysis technique. The statistical significance thresholds for differentially expressed lncRNA (DElncRNA) and mRNA (DEmRNA) samples were determined to be *p* < 0.05 and |log2FC| >1. Using these screening conditions, we identified the differential expression of lncRNAs and mRNAs between OSA patients and healthy controls. To reveal the sample specificity of differentially expressed lncRNA and mRNA, we utilized volcano plots and the “Pheatmap” package in R software (Khomtchouk et al., 2014) to conduct supervised hierarchical clustering based on the Euclide distance of the lncRNA and mRNA in the samples (Mielke and Berry, 2003; Bien and Tibshirani, 2011). Herein, P.Adjustp < adjustP & logFC > logFoldChange is an up-regulating gene, and P.Adjustp < logFC <(-log Fold Change) is a down-regulating gene.

### 2.7 Target gene prediction of differentially expressed miRNA

Identification of target genes is crucial for defining the function of miRNA. Due to the lack of miRNA information, the miRcode database (Jeggari et al., 2012) was utilized to predict the DElncRNA-targeted miRNA. Then, we predicted the target genes of differentially expressed miRNA using the mirtarBase (Chou et al., 2018), miRDB (Chen and Wang, 2020), and TargetScan (Lewis et al., 2003) databases. To improve the accuracy of miRNA prediction, we chose miRNA with common target genes across three databases.

### 2.8 Construction of OSA-related lncRNA-miRNA-mRNA network

The lncRNA that compete with miRNA for binding, miRNA of common target genes, and differential mRNA were incorporated into the ceRNA network, which was then visualized using Cytoscape software (Shannon et al., 2003) (version 3.8.2; http://cytoscape.org), resulting in the lncRNA-miRNA-mRNA ceRNA network diagram.

### 2.9 GO and KEGG functional enrichment analysis

For further analysis of the three domains of potential cell component (CC), molecular function (MF), and biological process (BP) of gene modules, the “ClusterProfiler” package (Yu et al., 2012) in R software was utilized for GO and KEGG pathway enrichment analysis of target genes. Each category describes the biological function of genes at varying depths. Utilizing KEGG pathway enrichment analysis, the enrichment degree of differential genes in pathways was analyzed. When *p* < 0.05, the GO term and KEGG pathway were designated as being enriched.

### 2.10 Construction of protein-protein interaction network and identification of hub genes

To further investigate the interactions between the corresponding genes in the ceRNA network, we constructed a PPI network using the Interaction Gene Retrieval Search Tool (STRING) (Szklarczyk et al., 2015) 11.0 (http://string-db.org/). It was assigned a confidence score greater than 0.15. Nodes in the PPI network results represent proteins, while lines represent protein interactions. We installed the Hubba plugin for Cytoscape (Chin et al., 2014) after identifying the hub genes among the common genes (http://hub.iis.sinica.edu.tw/cytohubba/). CytoHubba is a visualization program that generates dense relationships using degree, tight centrality, and moderate centrality algorithms. Using CytoHubba, the central gene in the ceRNA network was identified. Then, the top 10 genes were extracted using the five hub gene screening methods of MCC, degree, EPC, closeness, and betweenness. The “venndiagram” package (Lam et al., 2016) of R software was used to create a Venn diagram, and the final hub gene was determined by the intersection of the Venn diagrams.

### 2.11 Expression level and correlation analysis of hub genes

To understand the expression levels of the final hub genes, we used a *t*-test to compare the differences between the normal group and the OSA group for the final hub genes. Then, to gain a better understanding of the relationship between hub genes, Pearson’s correlation analysis was utilized, and the “Corrplot” package was used to visualize the results (Zhang et al., 2021a).

### 2.12 Verification of hub gene expression level

To validate the differential expression of hub genes between the OSA group and the normal group, we used the other three OSA patients as the internal validation cohort and the GSE135917 data set downloaded from the GEO data frame as the external validation cohort and then extracted the expression data from both sets of data. First, enter the search term “obstructive sleep apnea” on the homepage of the gene expression database (GEO) (http://www.ncbi.nlm.nih.gov/geo) for retrieval; the only allowed species is “*Homo sapiens*”; the data type is “expression profiling by array”. The dataset (GSE135, 917) was selected and queried from the GEO database, and then the platform file (GPL6244-17, 930) and matrix file (GSE135, 917) were downloaded. The GSE135917 dataset contains 8 normal patients and 10 OSA patients. This dataset also includes gene expression samples from 48 OSA patients receiving treatment. The *t*-test was utilized to compare the differences between the two groups, and the R packages “Ggplot2” (Zhang et al., 2021b) and “RColorBrewer” (Jędroszka et al., 2017) were utilized to visualize the results. *p* < 0.05 was considered statistically significant.

### 2.13 Construction of a genomic model based on predictor selection

To further screen the hub genes associated with a high risk of OSA, we used the “Glmnet” package in the R software to conduct the least absolute shrinkage and selection operator (LASSO) logistic regression to reduce the dimension of the data and determine the best prediction characteristics of the training cohort (Friedman et al., 2010). Then, the genes with non-zero coefficient characteristics in the LASSO regression model were chosen, and the “Rms” package in the R software was used to develop nomograms for them to identify patients at risk for OSA (Zheng et al., 2021).

### 2.14 Verification of nomogram

The nomogram is bootstrapped (1,000 bootstrap samples) in order to calculate the relative corrected C-index, which is used to evaluate the nomogram’s discrimination (Wolbers et al., 2009). The C-index ranges from 0.5 to 1.0, with 0.5 representing random chance and 1.0 representing complete discrimination (Wolbers et al., 2009). Medcalc software was used to evaluate the diagnostic value of the OSA nomogram using receiver operating characteristic (ROC) curves, and internal and external validation cohort ROC curves were used for further validation.

### 2.15 Construction of core ceRNA subnetworks for key genes

We remapped the validated key genes and their associated lncRNA and miRNA into ceRNA networks, which were visualized using the Cytoscape software.

### 2.16 Statistical methods

For correlation analysis, SPSS 26.0 statistical software, R 4.0.5 software, and Medcalc software were utilized. Counting data were expressed as frequency, while measurement data were expressed as mean ± standard deviation. *t*-test was used to analyze measurement data, while the chi-square test was used to analyze counting data. *p* < 0.05 was considered statistically significant.

## 3 Results

### 3.1 Basic characteristics of patients

In our study, we included 6 OSA patients and 3 healthy subjects, with 3 of them serving as the normal group (BMI: 22.77 ± 2.15), 3 obese OSA patients serving as the training cohort (BMI: 22.77 ± 2.15 vs. 31.40 ± 1.18, *p* = 0.004), and 3 non-obese OSA patients serving as the internal validation cohort (BMI: 22.77 ± 2.15 vs. 25.00 ± 1.06, *p* = 0.181). The AHI of the normal group was 1.53 ± 1.20 (times/hour), the obese group AHI was 89.93 ± 44.04 (times/hour), and the non-obese group AHI was 47.13 ± 22.01 (times/hour). In addition, the NoSAS score for the normal group was (4.00 ± 0.00), for the obese OSA group it was (13.00 ± 0.00), and for the non-obese OSA group it was (9.00 ± 1.73). The experimental group (obese OSA and non-obese OSA) consisted of patients with a high risk of OSA. There were almost no statistically significant differences between the three groups in terms of age, blood glucose, total cholesterol, triglyceride, high-density lipoprotein, and low-density lipoprotein (*p* > 0.05), and all patients were male, so they were well-matched. OSA patients had a larger neck circumference and waist circumference, and lower minimum oxygen saturation (Min-NOX) and mean oxygen saturation (Mean-NOX), and the difference between them was statistically significant (*p* < 0.05) (Table 1).

**TABLE 1:** Clinical characteristics of the study subjects.

	Training cohort			Internal validation cohort		
	Normal group	Obesity OSA	*P*	Normal group	Non-obese OSA	*P*
Number	3	3	—	3	3	—
Male	3	3	—	3	3	—
Age (years)	33.00 ± 4.36	32.00 ± 1.00	0.718	33.00 ± 4.36	31.00 ± 3.61	0.573
BMI(Kg/m2)	22.77 ± 2.15	31.40 ± 1.18	0.004	22.77 ± 2.15	25.00 ± 1.06	0.181
NC(cm)	37.67 ± 0.58	44.33 ± 3.51	0.032	37.67 ± 0.58	42.67 ± 0.58	<0.001
WC(cm)	84.67 ± 5.03	104.67 ± 8.50	0.025	84.67 ± 5.03	93.33 ± 3.79	0.076
SBP(mmHg)	120.33 ± 9.24	134.67 ± 4.93	0.077	120.33 ± 9.24	128.33 ± 10.97	0.389
DBP(mmHg)	72.67 ± 4.04	85.67 ± 4.16	0.018	72.67 ± 4.04	82.67 ± 8.50	0.140
HR(times/min)	68.00 ± 5.29	80.33 ± 16.62	0.288	68.00 ± 5.29	85.33 ± 5.51	0.017
NoSAS(points)	4.00 ± 0.00	13.00 ± 0.00	—	4.00 ± 0.00	9.00 ± 1.73	0.007
Blood sugar(mmol/L)	5.08 ± 0.68	5.21 ± 0.37	0.795	5.08 ± 0.68	5.37 ± 0.51	0.584
CHO(mmol/L)	3.72 ± 0.67	4.16 ± 0.64	0.450	3.72 ± 0.67	5.18 ± 0.55	0.043
TG(mmol/L)	0.85 ± 0.09	1.49 ± 0.62	0.151	0.85 ± 0.09	3.02 ± 1.94	0.126
HDL-C(mmol/L)	0.89 ± 0.19	0.95 ± 0.18	0.720	0.89 ± 0.19	1.20 ± 0.13	0.074
LDL-C(mmol/L)	2.73 ± 0.57	3.11 ± 0.66	0.492	2.73 ± 0.57	3.75 ± 0.78	0.141
AHI(times/hour)	1.53 ± 1.20	89.93 ± 44.04	0.025	1.53 ± 1.20	47.13 ± 22.01	0.023
Mean-NOX(%)	97.33 ± 0.58	91.67 ± 1.15	0.002	97.33 ± 0.58	94.67 ± 0.58	0.005
Min-NOX(%)	89.00 ± 6.08	59.33 ± 15.95	0.040	89.00 ± 6.08	73.67 ± 5.51	0.032

BMI, body mass index; NC, Neck circumference; WC, Waist circumference; SBP, systolic blood pressure; DBP, diastolic blood pressure; HR, Heart rate (times/min); CHO, total cholesterol; TG, triglycerides; HDL-C, high density lipoprotein; LDL-C, low density lipoprotein; AHI, apnea hypopnea index, mean NOx, average blood oxygen saturation, min; NOx, minimum blood oxygen saturation; OSA, obstructive sleep apnea, *P*: *p*-value.

### 3.2 Differential expression of lncRNA and mRNA between the OSA group and normal group

**FIGURE 1:**
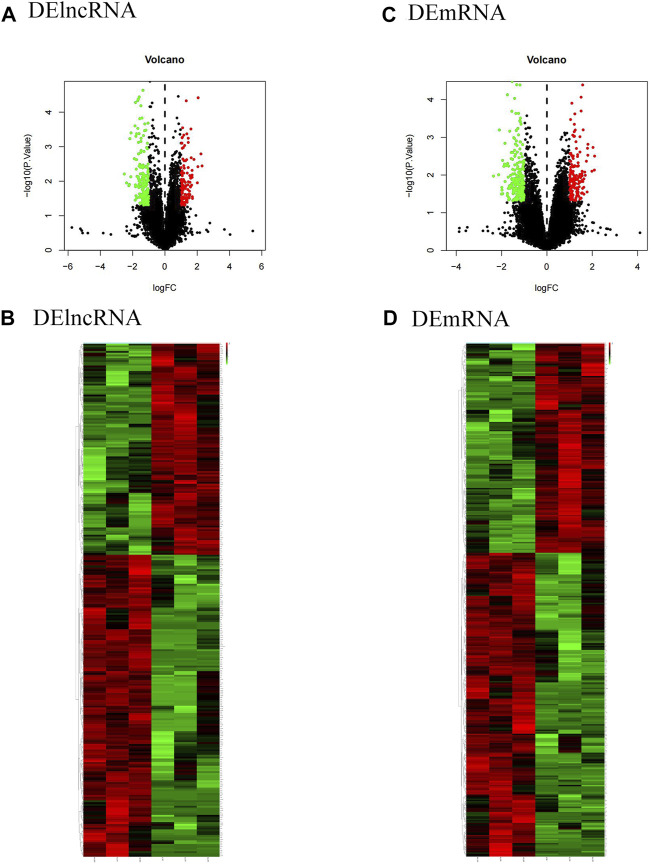
Expression analysis of DElncRNA and DEmRNA in the training cohort (volcano map and cluster heat map). **(A)**and **(C)** show the volcano plots of DElncRNA and DEmRNA, respectively. Red dots represent upregulated expression, and blue dots represent downregulated expression; **(B)** and **(D)** represent the clustering heatmaps of DElncRNA and DEmRNA, respectively. Red stripes show upregulated expression, while blue stripes show downregulated expression. DElncRNA: differentially expressed lncRNA; DEmRNA: differentially expressed mRNA.

To identify the differentially expressed lncRNA and mRNA between the OSA group and the normal group, the total RNA of 3 normal groups and 3 OSA groups was analyzed using microarrays. The microarray analysis revealed that lncRNA and mRNA were altered in the OSA group in comparison to the normal group. According to the screening criteria, 282 differential lncRNA, including 166 upregulated and 116 downregulated lncRNA, and 380 differential mRNA, including 225 upregulated and 155 downregulated mRNA, were screened. All gene expressions in the dataset were represented as volcano maps and cluster heat maps (Figure 1). Each point on the volcanic map represents a gene, with blue points representing genes with low expression and red points representing genes with high expression. The cluster heatmap displayed the differentially expressed lncRNA and mRNA between the OSA group and the normal group.

### 3.3 Construction of OSA-related lncRNA-miRNA-mRNA network

**FIGURE 2:**
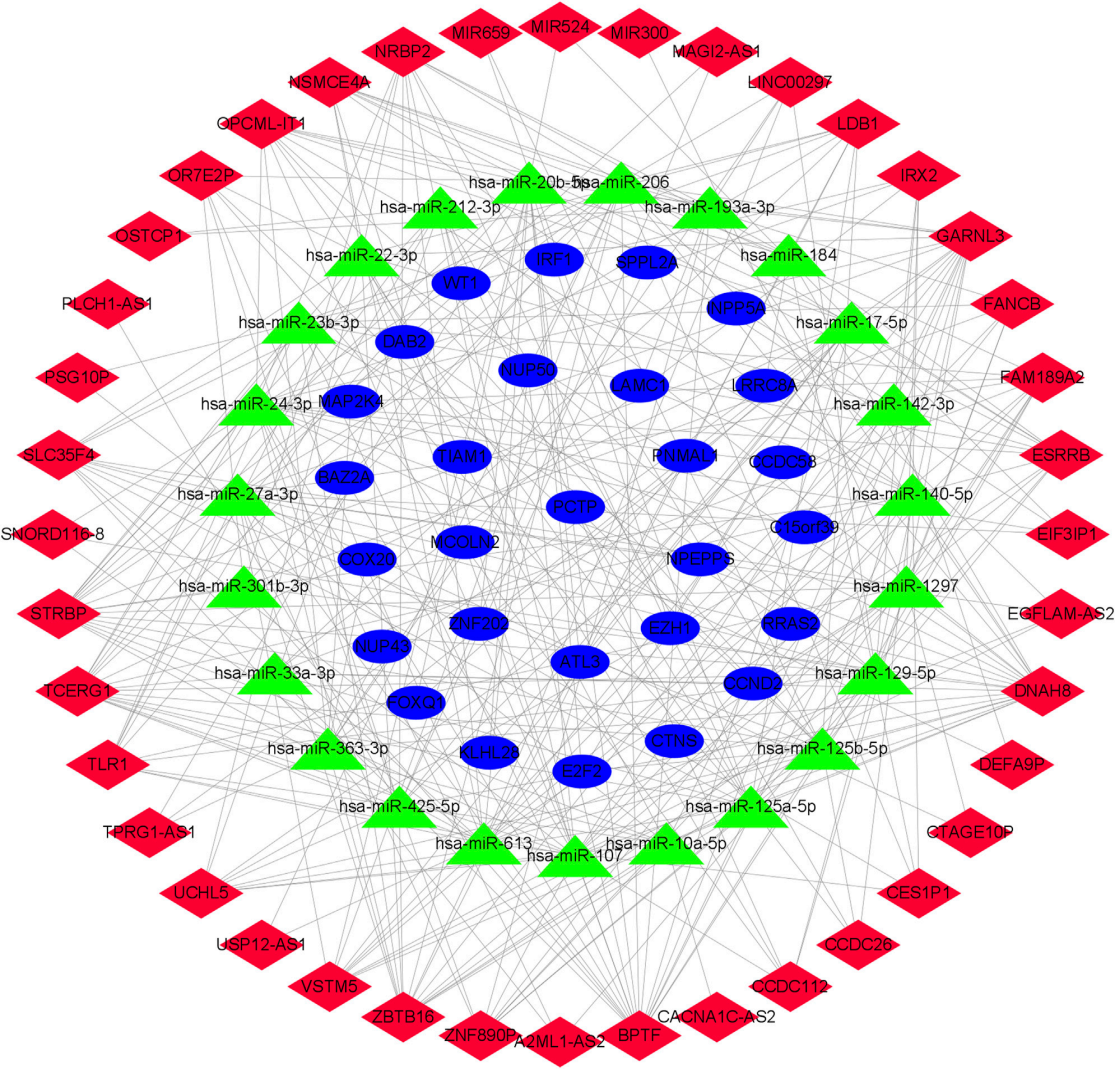
Network diagram of lncRNA-miRNA-mRNA. The construction of the ceRNA network includes 40 DElncRNA, 23 predicted miRNA and 28 DEmRNA. Blue circles, green triangles and red diamonds represent DEmRNA, miRNA and DElncRNA, respectively. DElncRNA: differentially expressed lncRNA; DEmRNA: differentially expressed mRNA; miRNA: microRNA.

We used the miRcode database to predict the 2,468 targeted miRNA of DElncRNA, followed by the mirtarBase, miRDB, and TargetScan databases to eliminate the 1935 miRNA of the common target genes of these three databases. Subsequently, lncRNAs (2,468) competing with miRNAs for binding, miRNAs of common target genes (1935), and differential mRNAs (380) were incorporated into the ceRNA network (Figure 2), and an OSA-related ceRNA network consisting of 292 miRNA-lncRNA and 41 miRNA-mRNA was developed,. The ceRNA network contained 23 predicted miRNA, 40 DElncRNA, and 28 DEmRNA.

### 3.4 Functional enrichment analysis

**FIGURE 3:**
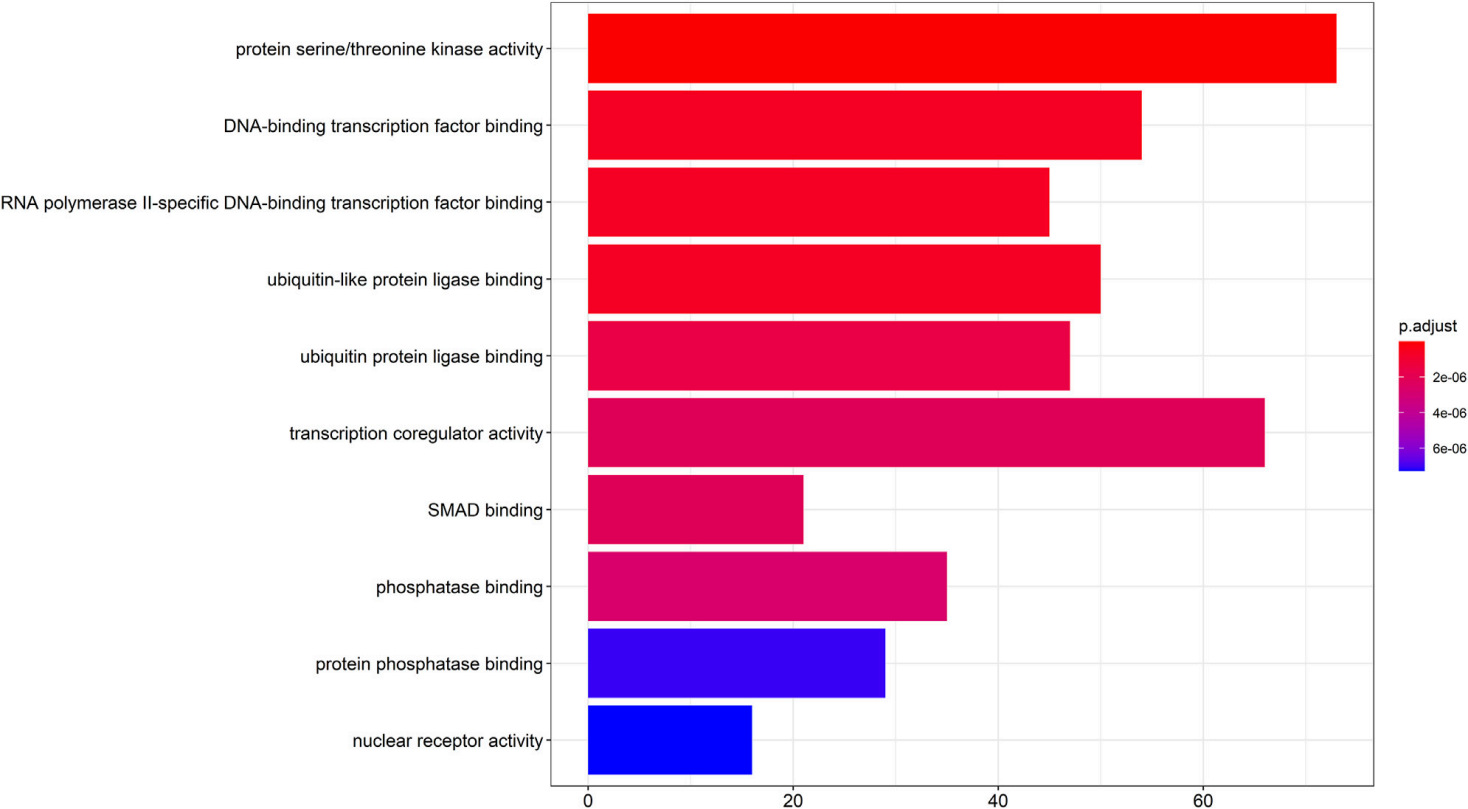
GO enrichment analysis diagram. GO enrichment analysis of target genes (*p* < 0.05 and *q* < 0.05), GO: Gene Ontology.

To gain a deeper understanding of the cellular processes mediated by target genes, a GO functional enrichment analysis was conducted to investigate the functional roles of their target genes in the fields of biological processes, cellular components, and molecular functions. Taking pvalueCutoff = 0.05 and qvalueCutoff = 0.05 as the criteria, we screened the enrichment analysis of the top 10 of *p*-value, which was mainly enriched in protein serine/threonine kinase (PKB, also known as Akt) activity, transcriptional co-regulatory activity, DNA-binding transcription factor binding, ubiquitin-like protein ligase binding, ubiquitin protein ligase binding, RNA polymerase II-specific DNA-binding transcription factor binding, phosphatase binding, protein phosphatase binding, SMAD binding and nuclear receptor activity (Figure 3).

**FIGURE 4:**
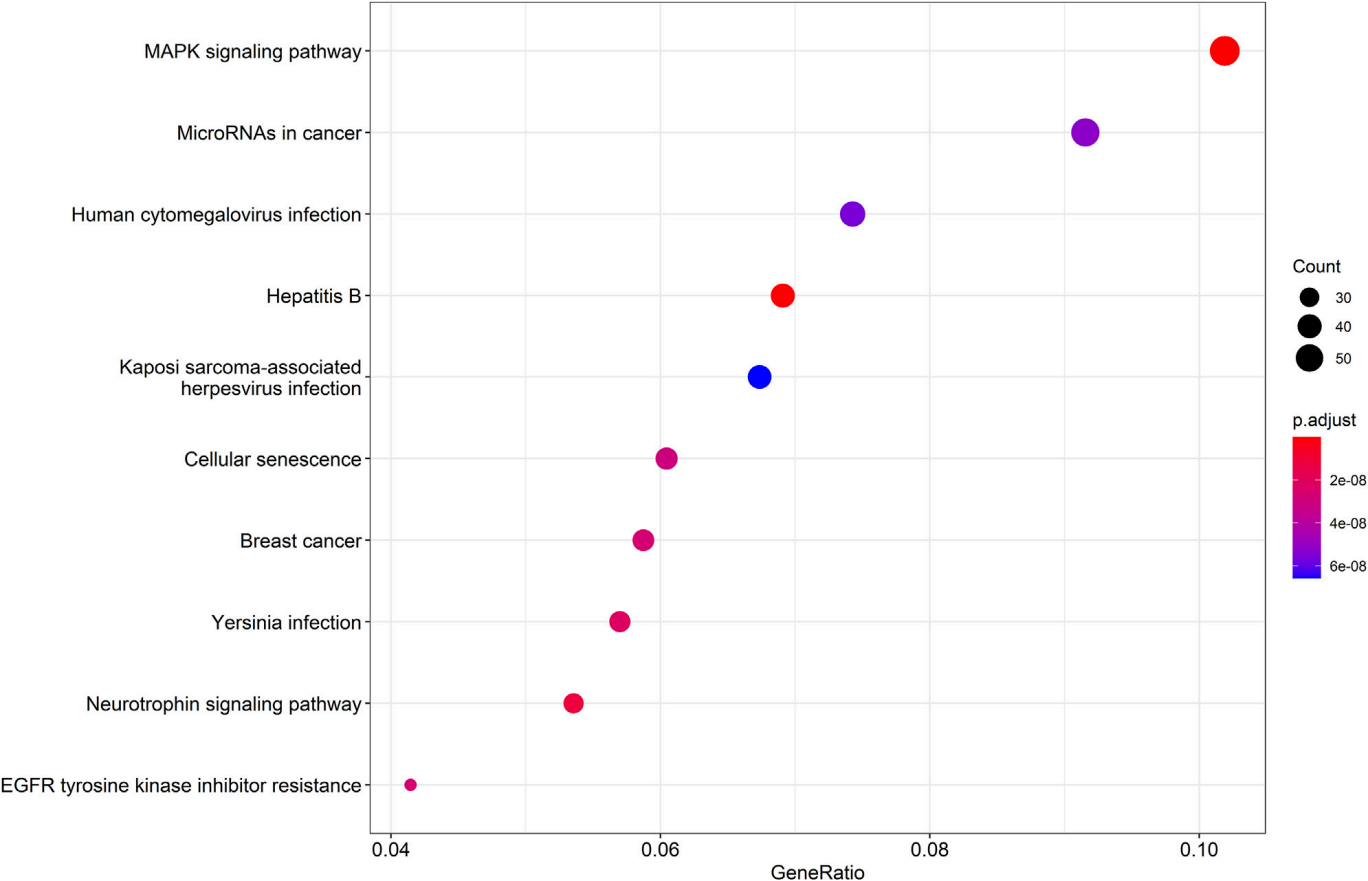
KEGG pathway enrichment analysis diagram. KEGG pathway enrichment analysis of target genes (*p* < 0.05 and *q* < 0.05), KEGG: Kyoto Encyclopedia of Genes and Genomes.

Then, a KEGG pathway enrichment analysis was performed to determine which pathways were significantly enriched in target genes. Using pvalueCutoff = 0.05 and qvalueCutoff = 0.05 as the criteria, we screened the pathway analysis of the top 10 of *p*-value, which was predominantly enriched in mitogen activated protein kinase (MAPK) signaling pathway, miRNA in cancer, human cytomegalovirus infection, Hepatitis B, Kaposi Sarcoma-associated herpesvirus infection, cellular senescence, breast cancer, *Yersinia* infection, neurotrophin signaling pathway, and EGFR tyrosine kinase inhibitor resistance (Figure 4).

### 3.5 PPI network analysis and hub gene selection

**FIGURE 5:**
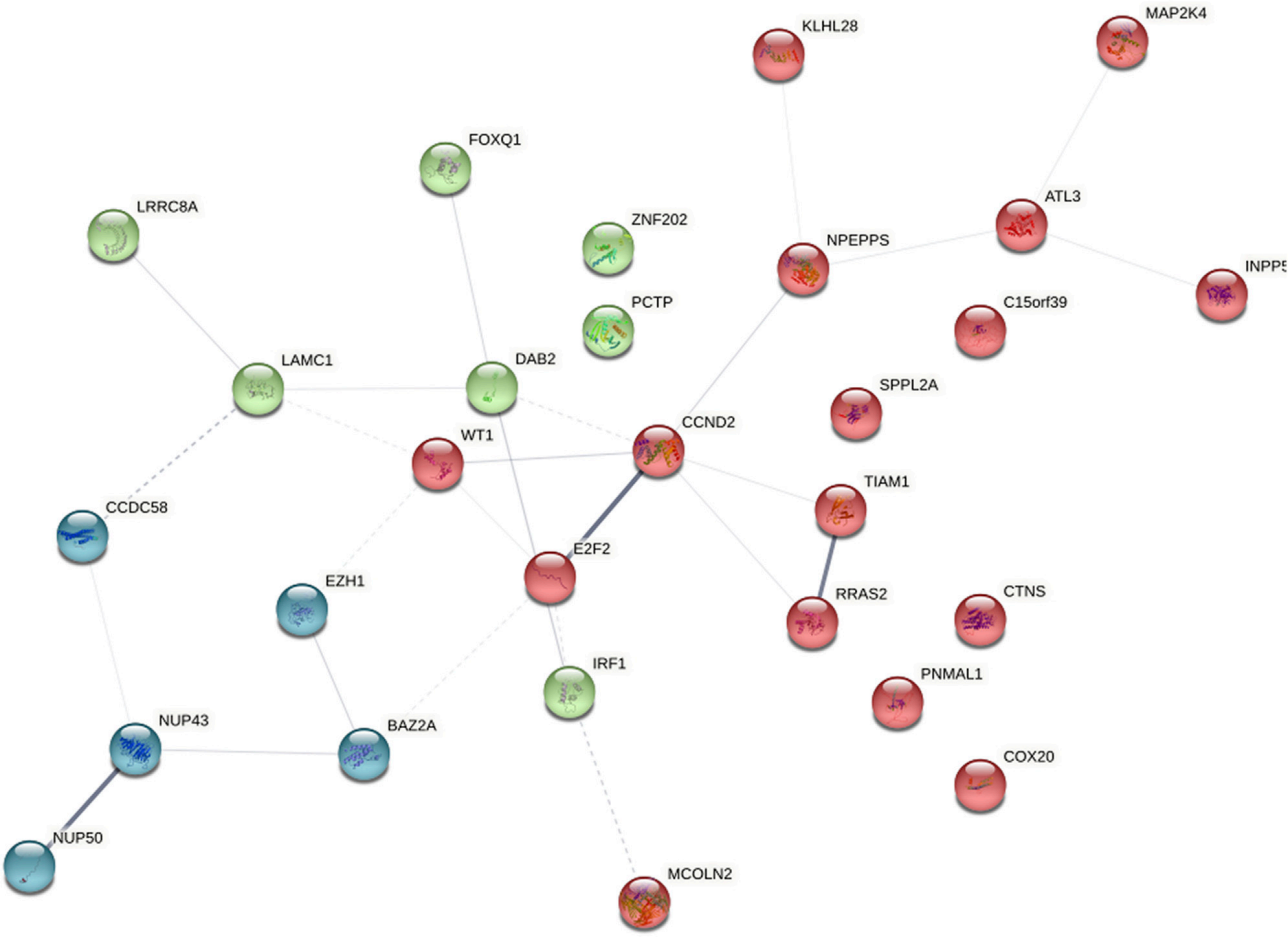
Key gene protein interaction (PPI) network.

**FIGURE 6:**
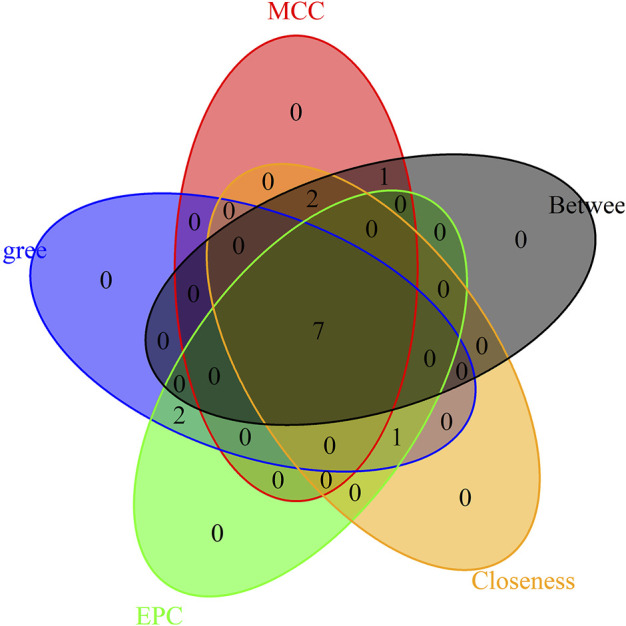
Venn diagram of five methods to screen the top ten hub genes.

To distinguish hub genes from common genes, we inserted the corresponding genes in ceRNA into the STRING database to build a PPI network (Figure 5). Subsequently, we uploaded the aforementioned PPI network relationship to Cytoscape and utilized its cytohubba plugin to identify hub genes. The MCC, EPC, Degree, Closeness, and Betweenness algorithms in Cytohubba were utilized to determine the top 10 hub genes. The scores of the five algorithms that screened the top 10 hub genes are shown in Table 2. To obtain a more robust hub gene, the top 10 hub genes identified by these five algorithms were intersected (Figure 6), resulting in the identification of 7 more robust hub genes, namely, CCND2, WT1, E2F2, IRF1, BAZ2A, LAMC1, and DAB2.

**TABLE 2:** Five methods of research subjects to screen the top 10 hub genes and their scores.

MCC		EPC		Degree		Closeness		Betweenness	
Name	Score	Name	Score	Name	Score	Name	Score	Name	Score
CCND2	6.000	CCND2	7.332	CCND	27.332	CCND2	11.750	CCND2	210.000
WT1	4.000	WT1	6.887	WT1	6.887	E2F2	10.500	NPEPPS	134.000
E2F2	4.000	E2F2	6.863	E2F2	6.863	WT1	10.417	E2F2	91.667
LAMC1	4.000	DAB2	6.341	DAB2	6.341	DAB2	10.417	DAB2	90.000
DAB2	4.000	LAMC1	6.145	LAMC1	6.145	LAMC1	9.900	LAMC1	79.333
IRF1	3.000	BAZ2A	5.687	BAZ2A	5.687	NPEPPS	9.367	ATL3	74.000
ATL3	3.000	IRF1	5.508	IRF1	5.508	IRF1	8.983	WT1	59.667
NPEPPS	3.000	EZH1	5.143	EZH1	5.143	BAZ2A	8.900	BAZ2A	55.667
BAZ2A	3.000	RRAS2	5.089	RRAS2	5.089	NUP43	8.150	NUP43	43.333
NUP43	3.000	TIAM1	4.991	TIAM1	4.991	EZH1	8.067	IRF1	42.000

OSA: obstructive sleep apnea, *P*: *p*-value.

### 3.6 Expression level and correlation analysis of hub genes

**FIGURE 7:**
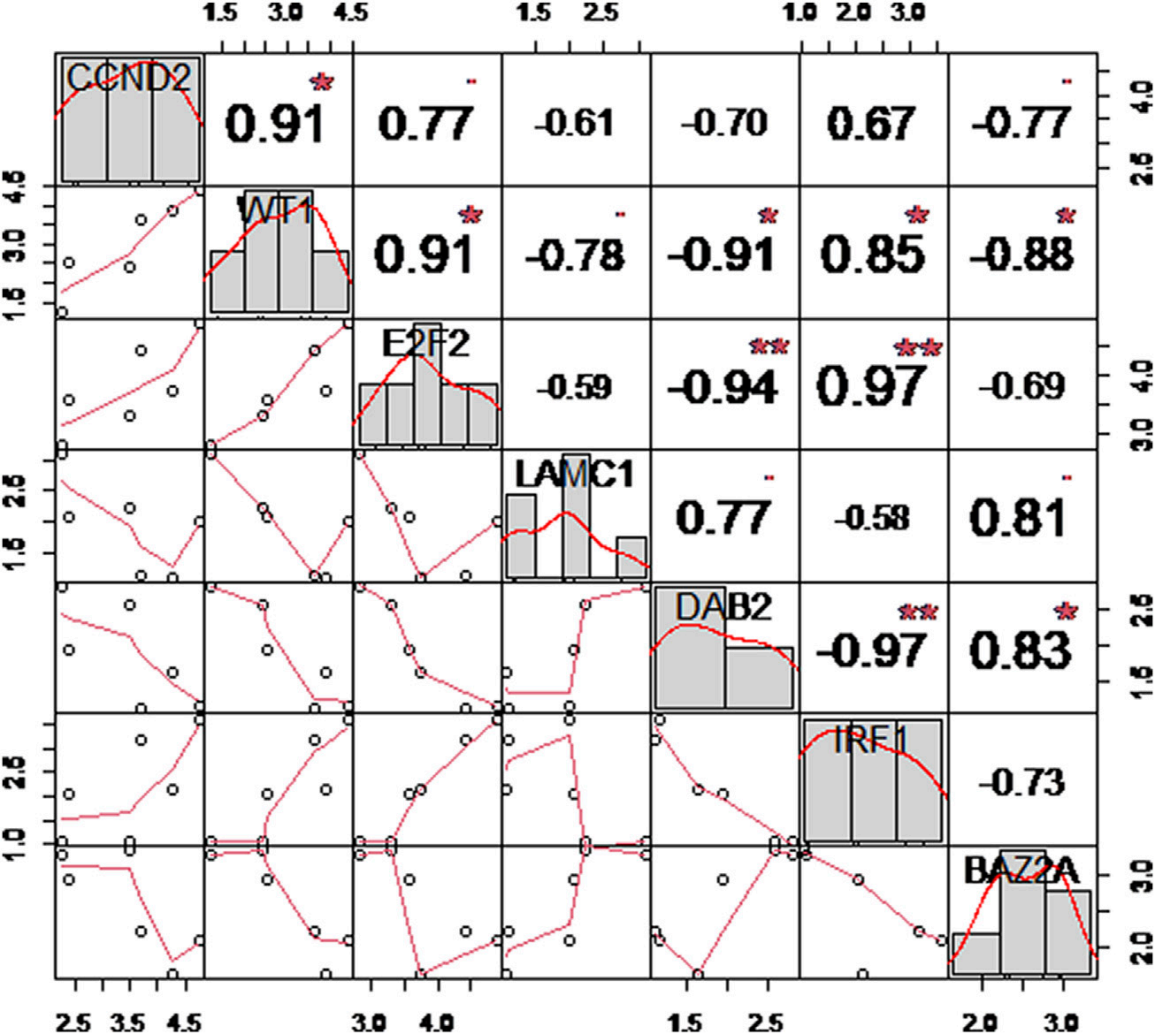
Correlation matrix analysis of robust hub genes. The distribution plot is shown on the diagonal; the lower left shows a bivariate scatterplot with a fitted line; and the upper right shows the correlation coefficient and significance level.

In order to comprehend the expression levels and correlations between these seven hub genes, we utilized the *t*-test to compare the variances of these seven hub genes between the normal and OSA groups. According to Table 3, the difference between the normal group and the OSA group was nearly statistically significant (*p* < 0.05). Then, we continued with the correlation matrix analysis of the seven hub genes. According to the classification of the Pearson correlation coefficient (r) (Hazra and Gogtay, 2016), the absolute values of 0–0.30, 0.30–0.50, 0.50–0.70, and 0.70–1.00 indicate “weak” correlation, “general” or “moderate” correlation, “good” correlation, and “strong” correlation, respectively. In addition, “r = 0” indicates “no correlation whatsoever” and “r = 1.00” indicates “complete correlation”. As shown in Figure 7, there was a good or strong correlation between the seven hub genes, with IRF1 being strongly positively correlated with E2F2 (r = 0.97) and strongly negatively correlated with DAB2 (r = −0.97), respectively.

**TABLE 3:** Expression levels of hub genes of the study subjects.

Name	Training cohort		Internal validation cohort				External validation cohort		
	Normal group	Obesity OSA	*P*	Normal group	Non-obese OSA	*P*	Normal group	OSA	*P*
CCND2	4.23 ± 0.53	2.70 ± 0.68	0.037	4.23 ± 0.53	3.66 ± 1.18	0.486	9.66 ± 0.55	10.72 ± 0.61	<0.001
WT1	3.98 ± 0.39	2.06 ± 0.73	0.016	3.98 ± 0.39	2.87 ± 0.67	0.069	6.49 ± 0.41	5.44 ± 0.19	<0.001
E2F2	4.34 ± 0.58	3.22 ± 0.39	0.051	4.34 ± 0.58	3.53 ± 1.41	0.411	6.90 ± 0.21	6.60 ± 0.18	<0.001
IRF1	2.98 ± 0.75	1.39 ± 0.57	0.043	2.98 ± 0.75	2.40 ± 1.48	0.578	7.58 ± 0.43	7.12 ± 0.32	0.002
BAZ2A	1.98 ± 0.31	3.20 ± 0.21	0.005	1.98 ± 0.31	1.92 ± 0.23	0.781	8.22 ± 0.30	8.12 ± 0.18	0.243
LAMC1	1.41 ± 0.52	2.46 ± 0.55	0.075	1.41 ± 0.52	1.80 ± 0.54	0.427	10.72 ± 0.35	10.56 ± 0.21	0.117
DAB2	1.32 ± 0.28	2.45 ± 0.44	0.021	1.32 ± 0.28	2.43 ± 2.08	0.412	9.89 ± 0.45	10.05 ± 0.37	0.326

OSA: obstructive sleep apnea, P: P value

### 3.7 Validation of hub gene expression levels

**FIGURE 8:**
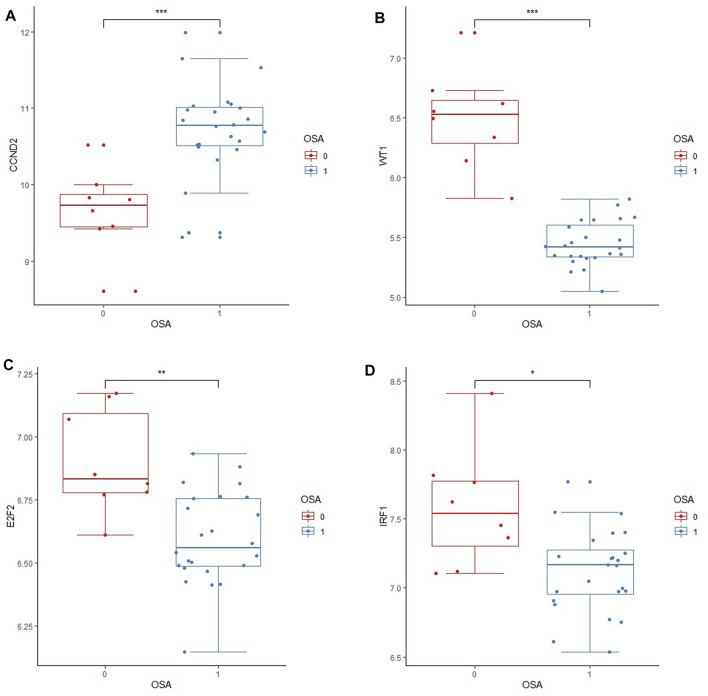
Boxplots of differential expression of key genes in the external validation cohort. External validation of four core genes in the GSE135917 dataset. “0”indicates the normal group; “1″indicates the OSA group. OSA stands for Obstructive Sleep Apnea. **(A)** The relative expression level of CCND2 between OSA and normal groups; **(B)** The relative expression level of WT1 between OSA and normal groups; **(C)** The relative expression level of E2F2 between OSA and normal groups; **(D)** The relative expression level of IRF1 between OSA and normal group. Data are presented as medians with interquartile ranges. *t*-test was used to compare relative expression levels between the two groups.

The downloaded GSE135917 dataset was preprocessed, and relevant information regarding the CCND2, WT1, E2F2, IRF1, BAZ2A, LAMC1, and DAB2 genes was then searched for. According to the expression profiling analysis of the GSE135917 data set, the expressions of CCND2, WT1, E2F2, and IRF1 in the OSA group were significantly decreased (*p* < 0.05), while there was no significant difference in BAZ2A, LAMC1, and DAB2 (*p* > 0.05) (Table 3). Figure 8 displays the results of the comparison between the two groups of CCND2, WT1, E2F2 and IRF1. According to the expression data of the internal validation cohort, although there was no statistically significant difference in the expression of CCND2, WT1, E2F2, and IRF1 (*p* > 0.05), this may be due to the difference caused by non-obese patients or the small sample size that did not achieve statistical significance. Nevertheless, according to the expression data from the external validation cohort, their expression differences were statistically significant (*p* < 0.05). The expression analysis of the four hub genes in the internal and external validation datasets was generally consistent with the training data set.

### 3.8 Construction of a genomic model based on predictor selection

**FIGURE 9:**
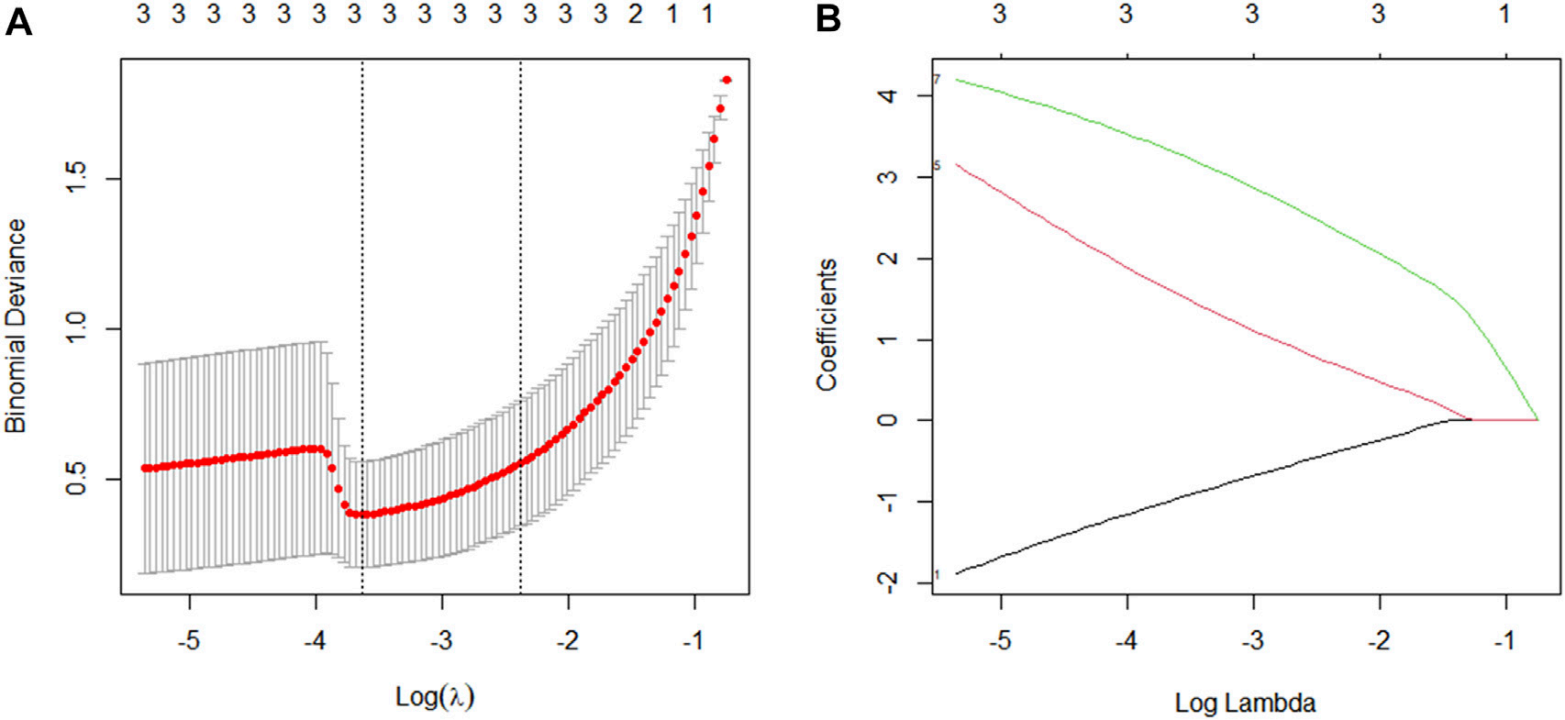
Factor selection using the Least Absolute Shrinkage and Selection Operator (LASSO) logistic regression model. **(A)**: The best parameter (lambda) selection in LASSO regression was selected using 10-fold cross-validation (through the minimum standard). Black vertical lines are drawn at the best values by using the minimum standard and one standard error of the minimum standard (1-SE standard). **(B)**: Three features of the lasso coefficient profile. Coefficient profiles are plotted according to the logarithmic (λ) series.

**FIGURE 10:**
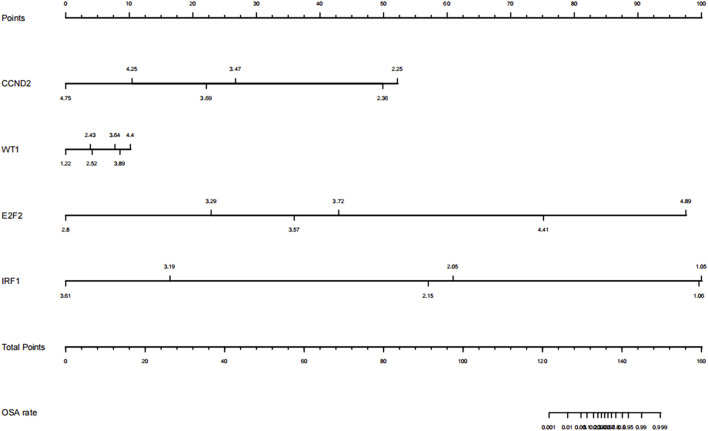
Nomogram for predicting obstructive sleep apnea syndrome. OSA: obstructive sleep apnea.

LASSO regression is appropriate for high-dimensional data regression. The compression coefficient is obtained by constructing a penalty function, and some compression coefficients are set to zero so that the most significant predictors can be extracted from the main data set and a more precise linear regression model can be developed (Friedman et al., 2010). In this study, a coefficient distribution curve was generated by calculating each subject’s risk score using a linear combination of factors weighted by the subject coefficient (Figure 9A). Figure 9B depicts the error plot for the cross-validation of the lasso regression model. The cross-validation error for the most regularized and parsimonious model was within 1 standard error of the minimum for 3 of the 7 variables. Four predictors (CCND2, WT1, E2F2, and IRF1) were ultimately chosen to develop an easy-to-use nomogram based on the expression level and correlation analysis of hub genes, the PPI network diagram, and its significance (Figure 10).

### 3.9 Verification of nomogram

**FIGURE 11:**
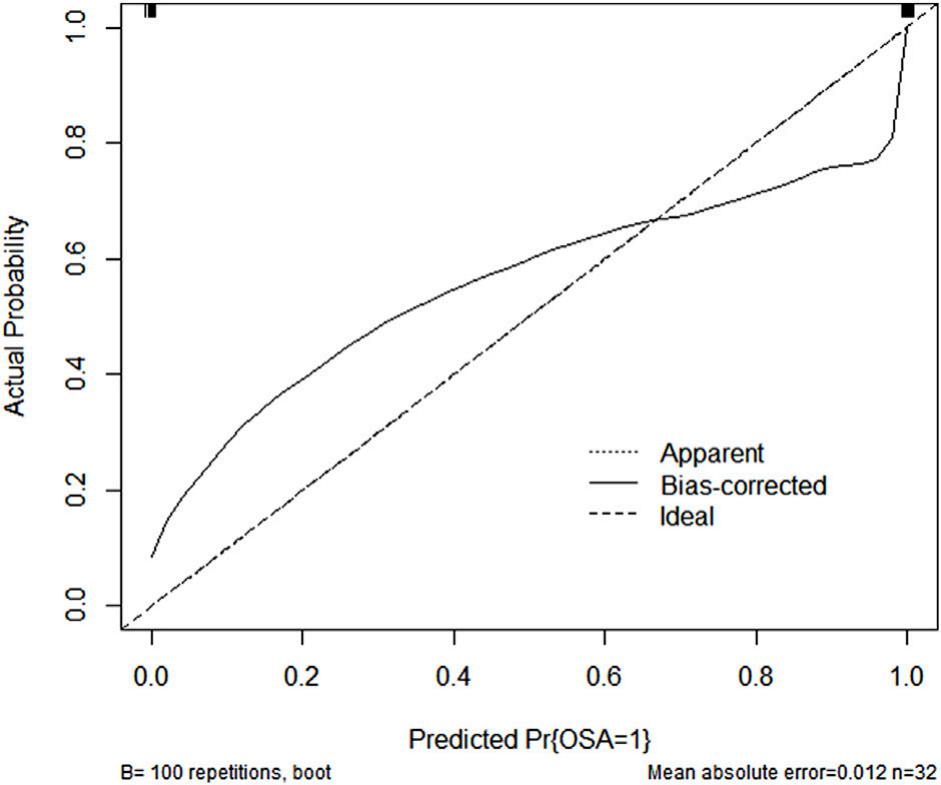
Calibration curve of nomogram in external validation cohort.

**FIGURE 12:**
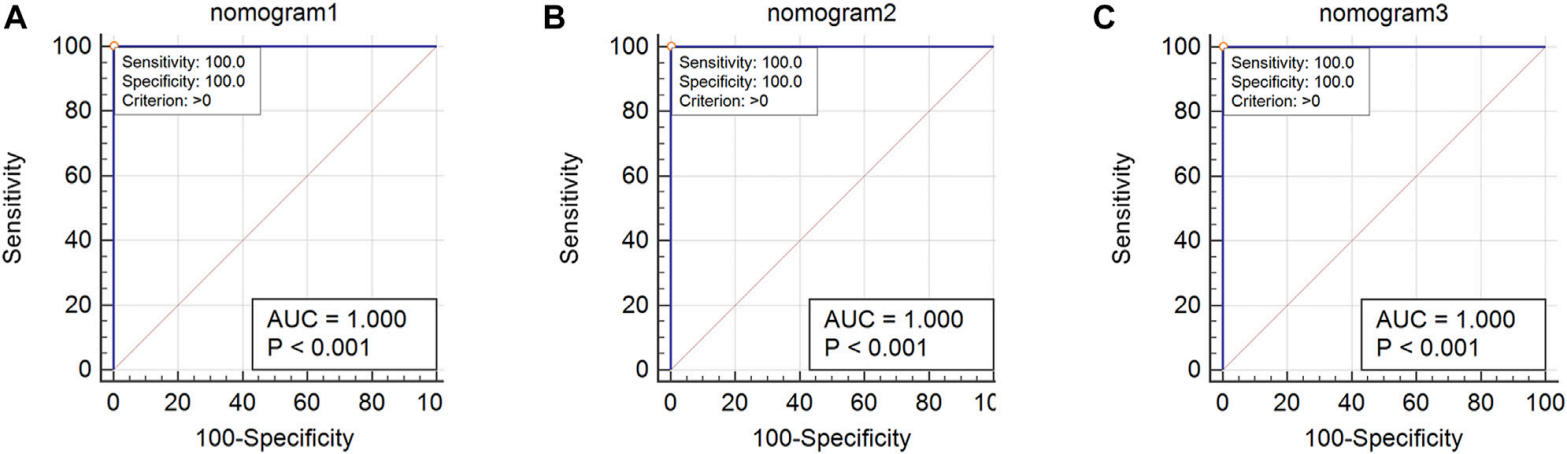
ROC curve of nomogram. **(A)**: Nomogram ROC of the training cohort; **(B)** Nomogram ROC of the internal validation cohort; **(C)** Nomogram ROC of the external validation cohort.

Using 1,000 bootstrap analyses, the validity of the nomogram was determined. In predicting OSA, the C-index of the nomogram for both the training cohort and the internal and external validation cohorts was 1, indicating that the model was sufficiently accurate; consequently, the model is appropriate for predicting OSA patients. Since there were fewer than 10 cases in the training and internal validation groups, no calibration plot could be generated. However, the calibration plot for external validation (Figure 11) revealed a relatively strong correlation between observed and predicted OSA. In addition, ROC curve analysis was used to evaluate OSA when the AHI cut-off value was 5 times/h based on the current nomogram. When the AUC of the nomogram was at the optimal cutoff point, regardless of whether the cohort was the training cohort, the internal validation cohort, or the external validation cohort, the ROC curve indicated that the diagnostic performance of the nomogram was improved (AUC = 1, AUC = 1, and AUC = 1), and their specificity and sensitivity were both 100% (Figure 12).

### 3.10 Construction of core ceRNA subnetworks for key genes

**FIGURE 13:**
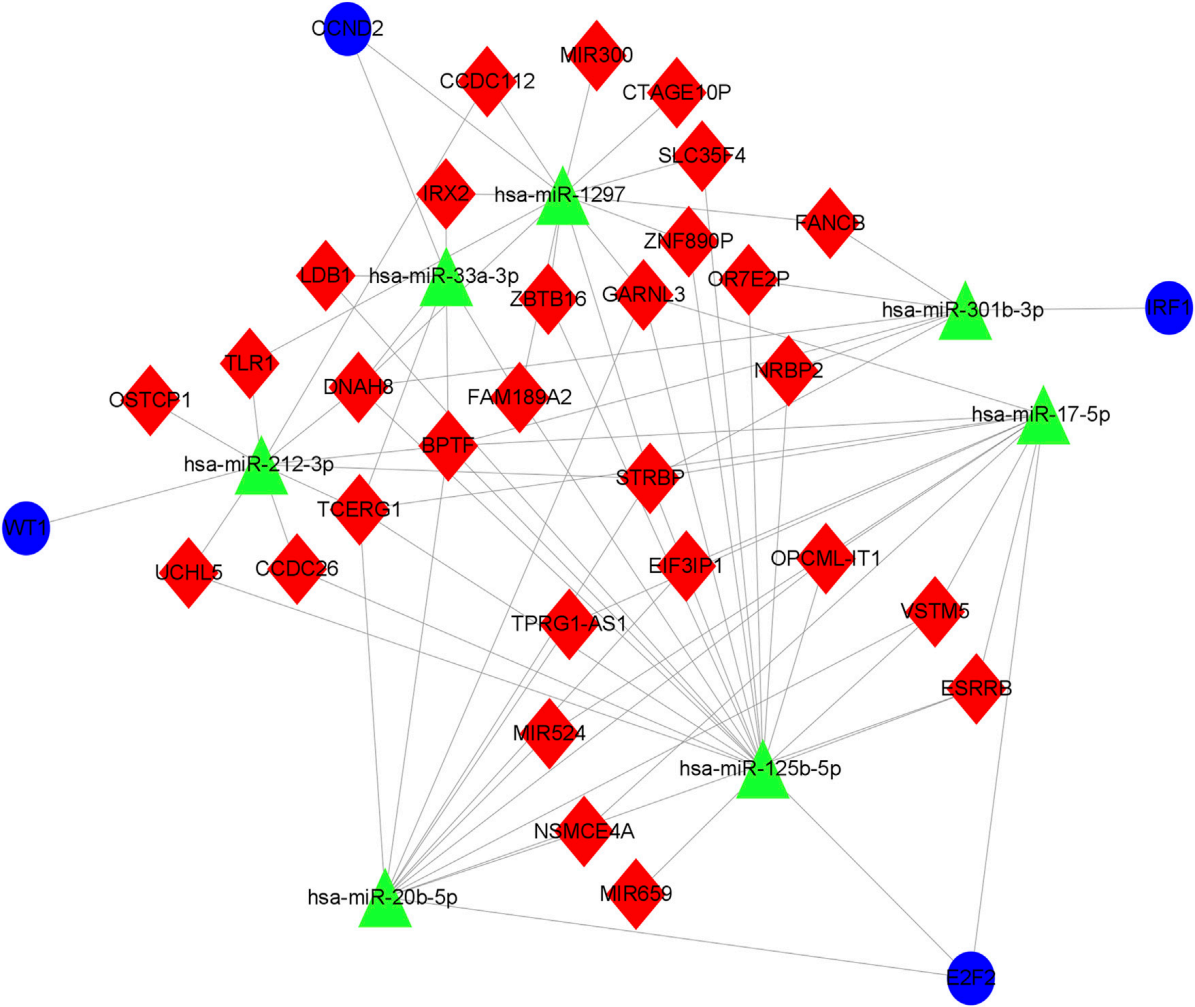
Core ceRNA sub-network diagram of key genes. The construction of the ceRNA network included 29 DElncRNA, 7 predicted miRNA and 4 DEmRNA. Blue circles, green triangles and red diamonds represent DEmRNA, miRNA and DElncRNA, respectively. DElncRNA: differentially expressed lncRNA; DEmRNA: differentially expressed mRNA; miRNA: microRNA.

Based on the ceRNA network, we remapped the four key genes of CCND2, WT1, E2F2, and IRF1, and related lncRNA and miRNA into the ceRNA network, thereby establishing a core ceRNA sub-network (Figure 13). It contained 74 miRNA-lncRNA and 7 miRNA-mRNA edges and 40 nodes [29 lncRNA (NSMCE4A, ZBTB16, TCERG1, UCHL5, ESRRB, DNAH8, FAM189A2, GARNL3, SLC35F4, ZNF890P, CCDC112, STRBP, IRX2, BPTF, TLR1, CTAGE10P, FANCB, NRBP2, LDB1, MIR659, MIR524, VSTM5, MIR300, CCDC26, TPRG1-AS1, EIF3IP1, OSTCP1, OPCML-IT1, and OR7E2P), 7 miRNA(hsa-miR-1297, hsa-miR- 33a-3p, hsa-miR-17–5p, hsa-miR-20b-5p, hsa-miR-125b-5p, hsa-miR-301b-3p, and hsa-miR-212–3p) and 4 mRNA(CCND2, WT1, E2F2 and IRF1)]. Figure 13 demonstrates that STRBP was the core lncRNA, capable of binding with hsa-miR-1297, hsa-miR-17–5p, hsa-miR-20b-5p, hsa-miR-125b-5p, hsa-miR-301b -3p, and hsa-miR-212–3p, which in turn affected four genes: CCND2, WT1, E2F2, and IRF1.

## 4 Discussion

Obstructive sleep apnea (OSA) is the most prevalent respiratory sleep disorder, affecting up to 1 billion individuals worldwide (Kendzerska et al., 2021). In addition, the patient’s compliance with the diagnosis and treatment of OSA is poor, which can easily lead to the disease’s progression, which will impose a heavy burden on society and present treatment challenges for physicians. In order to prevent the progression of OSA, effective screening technologies, accurate diagnosis, and treatment remain crucial. Recent studies have highlighted the regulatory role of lncRNA as ceRNA in the development and occurrence of chronic intermittent hypoxia (Ge et al., 2019; Zhang et al., 2020a; Hu et al., 2021). We constructed an lncRNA-related ceRNA network based on the results of the OSA ceRNA chip to identify new targets with potential diagnostic or therapeutic value for OSA, and then validated the new targets.

In this study, we successfully identified 282 DElncRNA and 380 DEmRNA through the differential expression of lncRNA and mRNA in order to reduce the error interference between the OSA group and the normal group. Initially, we matched the key information of the two groups in order to reduce the error interference between the OSA group and the normal group. Combining lncRNA that compete with miRNA for binding, miRNA of common target genes, and differential mRNA yielded a ceRNA network. The target genes in the ceRNA network were then analyzed for enrichment in GO terms and KEGG pathways. GO enrichment analysis revealed that target genes were primarily enriched in protein serine/threonine kinase activity, transcriptional co-regulatory activity, DNA-binding transcription factor binding, ubiquitin-like protein ligase binding, ubiquitin protein ligase binding, and so on. Protein serine/threonine kinase activity was, without a doubt, the most important GO pathway. PKB consists of three widely expressed isoforms (PKBα, PKBβ, and PKBγ; also known as Akt1, Akt2, and Akt3, respectively), and PKBβ may be an important mediator in the insulin signaling transduction pathway (Lawlor and Alessi, 2001). Analysis of KEGG pathway annotations revealed that target genes were predominantly involved in the MAPK signaling pathway, miRNA in cancer, human cytomegalovirus infection, Hepatitis B and Kaposi sarcoma-associated herpesvirus infection, etc. However, the MAPK signaling pathway was the pathway with the greatest enrichment. MAPK is a ubiquitous family of proline-directed protein serine/threonine kinases that are required for the sequential transduction of biological signals from the cell membrane to the nucleus (Broom et al., 2009). OSA-induced intermittent hypoxia has been reported to excessively and persistently activate the MAPK signaling pathway (Zhao et al., 2016). Some studies have also demonstrated that at the cellular level, chronic intermittent hypoxia alters the equilibrium between the phosphatidylinositol 3-kinase (PI3K)-dependent insulin signaling pathway, which regulates the production of endothelial nitric oxide (NO), and the activation of the mitogen-activated protein kinase (MAPK)-dependent insulin signaling pathway, which regulates the secretion of vasoconstrictor endothelin-1 (ET-1), thus affecting vascular endothelial dysfunction (Sharma et al., 2018). Clearly, protein serine/threonine kinase activity plays a pivotal role in the MAPK signaling pathway during the progression of OSA disease.

Then, we constructed a network of lncRNA-related ceRNAs and identified 28 hub genes. Then, a PPI network was created, and the cytohubba plug-in was utilized to identify 7 stable hub genes (CCND2, WT1, E2F2, IRF1, BAZ2A, LAMC1, and DAB2). IRF1 was strongly positively correlated with E2F2 (r = 0.97), and IRF1 was strongly negatively correlated with DAB2 (r = −0.97), according to the results of the correlation analysis. In addition, the levels of expression of these seven hub genes were validated using both internal and external validation datasets. LASSO regression was then applied to the seven hub genes. In conjunction with the expression level and correlation analysis of hub genes, as well as the PPI network diagram and its significance, the number of candidate variables was reduced to 4 potential predictors (CCND2, WT1, E2F2, and IRF1). It has been reported that CCND2 regulates cell proliferation by binding to cyclin-dependent kinase 4 (CDK4) or cyclin-dependent kinase 6 (CDK6) to form a complex required for the G1/S cell cycle (Chermuła et al., 2019). Furthermore, it has been reported that CCND2 is one of the most important biomarkers of endothelial dysfunction (Zhu et al., 2021). WT1 is a transcription factor that is unique among transcription factors because it functions as both a tumor suppressor and an embryonic development regulator (Krueger et al., 2019). It has been demonstrated that the expression of WT1, which is upregulated by hypoxia in endothelial cells, and the proliferation of endothelial cells are regulated by WT1 (Duim et al., 2015). E2F2, a member of the E2F family, regulates the cell cycle by inhibiting or activating cell cycle regulators, such as cyclins, cyclin-dependent kinases (CDKs), and checkpoint regulators (Li et al., 2014). Experiments have demonstrated that E2F1 inhibits angiogenesis and endothelial cell proliferation following ischemic injury by suppressing the expression of pro-angiogenic cytokines, vascular endothelial growth factor, and placental growth factor (Zhou et al., 2013). Interferon regulatory factor-1 (IRF1), a member of the IRF family of transcription factors, regulates gene expression during inflammation, immune response, cell proliferation, cell cycle progression, T cell differentiation, and DNA damage (Huang et al., 2019). Studies indicate that hypoxia can regulate the transcription of KPNA2 by simultaneously increasing the expression of its positive regulator E2F1 and inhibiting the expression of its negative regulator IRF1 (Huang et al., 2019). Clearly, these four predictors are associated with hypoxia, and intermittent hypoxia is one of the underlying causes of OSA. Consequently, CCND2, WT1, E2F2, and IRF1 target genes are all associated with OSA, and their inclusion in the model is reasonable.

Nomogram is a risk prediction tool that has been used for decades in medicine. By combining important predictors to predict clinical events and outcomes, it has been widely used to predict the risk and prognosis of various diseases (Chen et al., 2021; Song et al., 2021). Zhu et al. (2020) constructed a nomogram using seven hub RNA (HMMR, RNF24, RAP2A, S100A10, ARL2, has-miR-326, and hsa-miR-421). Consistently, the calibration curve demonstrated that the risk prediction model for hepatocellular carcinoma based on seven hub RNA had an adequate predictive effect. Song and Fu, 2019 developed a nomogram that included the target gene CXCR5, age, and stage. In the training set, the AUC values for the nomogram’s ability to predict the 3-year and 5-year overall survival of colorectal cancer were 0.749 and 0.805, respectively, whereas, the corresponding values in the validation set were 0.706 and 0.779, respectively. Shi et al. (2020) established a nomogram that included waist-to-hip ratio, smoking status, BMI, uric acid, Homeostasis Model Assessment 2 Insulin Resistance Index (HOMA2-IR), and history of fatty liver, and the AUC for distinguishing non-OSA patients from OSA patients was 0.855. Luo et al. (2015) established a nomogram that incorporated numerous subjective and objective variables (disease duration, smoking status, sleep difficulties, lack of energy, and waist circumference), and its discrimination accuracy for non-OSA, moderate-to-severe OSA, and severe OSA was 83.8%, 79.9%, and 80.5%, respectively. Based on these prediction models, it is evident that, regardless of whether the research is fundamental or clinical, nomograms are generally effective at predicting disease, which provides a foundation for identifying reliable targets. Verification of the nomogram is crucial for avoiding overfitting and determining generality (Iasonos et al., 2008). In our study, after proper calibration, the validation cohort’s calibration curve revealed that the actual occurrence probability was relatively close to the predicted occurrence probability. In addition, the training cohort, internal validation cohort, and external validation cohort all have C-index and AUC values of 1, indicating that the model is sufficiently accurate and diagnostically efficient. Thus, the validity of our nomogram has been established.

The nomogram has been recognized as a reliable tool for quantifying disease risk based on multivariate modeling procedures (Luo et al., 2015), and the nomogram constructed by target genes such as CCND2, WT1, E2F2, and IRF1 has been demonstrated to be robust; consequently, CCND2, WT1, E2F2, and IRF1 may be reliable OSA targets. In order to gain a deeper understanding of the regulatory mechanisms of these four target genes, we remapped them back into the ceRNA network, establishing a core ceRNA sub-network to search for important lncRNA or miRNA, or even lncRNA-miRNA-mRNA regulatory axes. The results demonstrated that STRBP was a core lncRNA that could bind competitively with hsa-miR-1297, hsa-miR-17-5p, hsa-miR-20b-5p, hsa-miR-125b-5p, hsa-miR-301b-3p, and hsa-miR-212-3p, thereby regulating the four genes CCND2, WT1, E2F2, and IRF1.

The ceRNA mechanism is a critical mode of regulation for cellular active metabolism and disease. STRBP is a sperm perinuclear RNA-binding protein that resides on chromosome 9q33, is widely expressed in lymph nodes, testis, and other tissues, and plays a crucial role in mammalian spermatogenesis (Zhang et al., 2020b). According to reports, STRBP can be detected in lung adenocarcinoma, breast cancer, and hematological malignancies (Zhang et al., 2020b). STRBP may be associated with body weight, according to studies (Wang et al., 2020). miRNA are commonly used in bioinformatics target prediction algorithms, and seed matching, sequence conservation, and thermodynamics of miRNA-mRNA interactions are commonly used to predict potential targets (Angerstein et al., 2012). miR-1297 inhibits KPNA2 in glioblastoma to negatively regulate metabolic reprogramming (Li and Yuan, 2020). According to previous studies, the CCND2 gene is a potential target of miR-1297, which inhibits the progression of colorectal cancer by inhibiting the transcription of CCND2 in colorectal cancer cells (Wang et al., 2017). It has been reported that miR-17-5p plays a role in the proliferation of pulmonary vascular smooth muscle cells, making it a potential new therapeutic target for the control of pulmonary hypertension (Yao et al., 2021).hsa-miR-17-5p may play a significant role in hypertrophic cardiomyopathy and is anticipated to serve as a diagnostic biomarker for this condition (Shi et al., 2019). Drobna et al. (2020) discovered that hsa-miR-20b-5p affected the expression of the tumor suppressor genes PTEN and BIM and regulated the survival of T-cell acute lymphoblastic leukemia cells *in vitro*. In addition, miR-20b-5p is predicted to regulate the TNFα signaling pathway, which supports the notion that diabetic retinopathy progression is primarily driven by retinal inflammation (Trotta et al., 2021). It has been reported that miR-125b-5p, a member of the miR-125 family, regulates the proliferation of differentiated tumor cells and may be a diagnostic biomarker for early cervical cancer and rheumatoid arthritis (Deping et al., 2021). Wu et al. (2020) discovered that the regulatory axis of hsa_circ_0000069/hsa-miR-125b-5p/CDKN2A may play a role in the occurrence and progression of cervical squamous cell carcinoma. miR-301b expression was induced by hypoxia in PrCa cell lines (DU145, PC-3, LNCaP) *in vitro*, resulting in increased autophagy and loss of radiosensitivity, thereby influencing the occurrence and progression of prostate cancer (Fort et al., 2018). Validation of cell lines and cell line-derived exosomes demonstrated that exosome-specific hsa-miR-301b-3p was upregulated in both eye cancer cell lines and their exosomes (Ravishankar et al., 2020). Previous research has demonstrated that the entire genome of hsa-miR-212-3p is downregulated in Alzheimer’s disease, with a more pronounced decrease in Alzheimer’s disease samples containing gray matter (Pichler et al., 2017). In the study by Cheng and Wang, 2020 lncRNA XIST regulates the expression of ASF1A and BRWD1 *via* miR-212–3p, influencing the occurrence and development of acute kidney injury. As far as we know, hypoxia is a condition of insufficient tissue oxygenation that plays an important role in numerous pathophysiologies, including embryonic development, high-altitude adaptation, inflammation, tissue repair, and tumor growth (Krueger et al., 2019), whereas chronic intermittent hypoxia can cause OSA (Zhou et al., 2021). In addition, OSA has been linked to cardiovascular disease, type 2 diabetes, Alzheimer’s disease, pulmonary hypertension, and kidney damage (Daulatzai, 2013; Abuyassin et al., 2019; Zhou et al., 2021). In conclusion, we hypothesize that lncRNA STRBP may compete with miRNA (hsa-miR-1297, hsa-miR-17-5p, hsa-miR-20b-5p, hsa-miR-125b-5p, hsa-miR-301b-3p, and hsa-miR-212-3p) for binding, thereby regulating the target genes of CCND2, WT1, E2F2, and IRF1, affecting the occurrence and development of OSA; however, the specific pathogenesis still warrants further investigation. Although these four key hub genes and related mechanism networks may not be specific and require further validation, they can still provide a new direction for the diagnosis and treatment of OSA in patients.

## 5 Advantages and limitations

There are advantages and limitations to this study. First, to the best of our knowledge, this may be the first study to construct a human plasma lncRNA-related ceRNA network, followed by the development of a predictive model for key hub genes and internal and external validation. The findings in this study provide a new perspective on the functional mechanism of OSA and theoretical support for the potential diagnostic and therapeutic targets. Nevertheless, our study has many limitations. First, we only compared ceRNA between OSA and normal plasma; however, it may differ between OSA severity levels and must be identified further. Second, the sample size used for analysis and validation is smaller than the sample size typically required for biomarker analysis, which may result in errors. Thirdly, the external validation is based solely on public databases, and our results require additional *in vivo* and *in vitro* validation. Therefore, we must conduct a prospective cohort study with a larger sample size to further confirm our position.

## 6 Conclusion

In conclusion, our findings indicate that protein serine/threonine kinase activity plays a crucial role in the MAPK signaling pathway during the progression of OSA disease. CCND2, WT1, E2F2, and IRF1 could be new OSA targets for diagnosis and treatment. Using these four key hub genes, we designed and validated a new nomogram to predict the risk of OSA patients that has sufficient performance and discrimination ability to serve as a basis for clinical decision-making. LncRNA STRBP may compete with miRNA (hsa-miR-1297, hsa-miR-17-5p, hsa-miR-20b-5p, hsa-miR-125b-5p, hsa-miR-301b-3p and hsa-miR-212-3p) for binding, thereby regulating the target genes of CCND2, WT1, E2F2 and IRF1, affecting the occurrence and development of OSA. In conclusion, despite the fact that our results are preliminary, these analyses provide a new direction for the pathogenesis of OSA; consequently, they may aid in the future translation of this study into clinical work.

## Data Availability

The original contributions presented in the study are publicly available. This data can be found here: https://www.ncbi.nlm.nih.gov/geo/. Accession number: GSE226379.

## Glossary

OSA: Obstructive sleep apnea
ceRNA: Competitive endogenous ribonucleic acid
ncRNA: Non-coding ribonucleic acid
miRNA: Micro-ribonucleic acid
lncRNA: Long non-coding ribonucleic acid
circRNA: Circular ribonucleic acid
inRNA: Intronic ribonucleic acid
siRNA: Small interfering ribonucleic acid
snoRNA: Small nucleolar ribonucleic acid
**piRN**A: Piwi-interacting ribonucleic acid
mRNA: Messenger ribonucleic acid
DElncRNA: Differentially expressed long non-coding ribonucleic acid
DEmRNA: Differentially expressed messenger ribonucleic acid
HIF-1: Hypoxia-inducible factor 1
BMI: Body mass index
AHI: Apnea hypopnea index
GO: Gene Ontology
KEGG: Kyoto Encyclopedia of Genes and Genomes
STRING: The Search Tool for the Retrieval of Interacting Genes
BP: Biological processes
CC: Cellular component
MF: Molecular function
PPI: Protein-protein interaction network
LASSO: Least absolute shrinkage and selection operator
Min-NOX: Minimum nocturnal oxygen saturation
Mean-NOX: Mean nocturnal oxygen saturation
AUC: Area under the curve
MAPK: Mitogen activated protein kinase
PKB: Protein kinase B
NC: Neck circumference
WC: Waist circumference
SBP: Systolic blood pressure
DBP: Diastolic blood pressure
HR: Heart rate
TG: Triglycerides
HDL-C: High density lipoprotein
LDL-C: Low density lipoprotein
